# An adolescent case of pancreatic tuberculosis after treatment of latent tuberculosis infection

**DOI:** 10.1111/ped.70127

**Published:** 2025-08-28

**Authors:** Yuya Nakano, Kenta Yamada, Koji Suzuki, Motoko Yasutomi‐Sakai, Motohiro Takeuchi, Yusei Ohshima

**Affiliations:** ^1^ Department of Pediatrics Fukui Red Cross Hospital Fukui City Fukui Japan; ^2^ Department of Pediatrics University of Fukui Yoshida‐gun Fukui Japan; ^3^ Division of Infectious Disease, Department of Pediatrics Hyogo Prefectural Kobe Children's Hospital Chuo‐ku Kobe Hyogo Japan; ^4^ Department of Pediatrics National Hospital Organization Tsuruga Medical Center Tsuruga City Fukui Japan; ^5^ Hyogo Prefectural Amagasaki General Medical Center Amagasaki Hyogo Japan

**Keywords:** adolescent, fine needle aspiration, latent tuberculosis infection, pancreatic neoplasm, pancreatic tuberculosis

Pancreatic tuberculosis (TB) is a rare form of extrapulmonary TB.^1^ We present a case of pancreatic TB requiring differentiation from pancreatic neoplasms.

A 13‐year‐old boy born in the Philippines presented with fever and back pain lasting 6 days. While living in the Philippines, his grandmother developed pulmonary TB. After immigrating to Japan at the age of 9 years, the pre‐entry tuberculosis screening test showed a positive T‐SPOT, and the chest X‐ray and chest computed tomography (CT) showed hilar lymph node calcification. Gastric juice smear revealed Gaffky 0 and *Mycobacterium tuberculosis* Polymerase chain reaction (PCR) was negative. In consultation with the Japan Anti‐Tuberculosis Association Research Institute and the Health Center, he received a 6‐month regimen of isoniazid (INH) for latent TB infection. On admission, his body temperature was 38.1°C, and localized tenderness was noted in both upper abdominal quadrants. Laboratory findings were as follows: white blood cell count, 9.48 × 10^9^/L (neutrophils, 70.8%; eosinophils, 0.4%; basophils, 0.5%; lymphocytes, 18.3%; and monocytes, 10%); platelets, 407 × 10^9^/L; C‐reactive protein, 7.76 mg/dL; aspartate transaminase, 112 IU/L; alanine transaminase, 224 IU/L; gamma‐glutamyl transferase, 225 IU/L; amylase, 723 IU/L; and P‐amylase, 716 IU/L. Abdominal ultrasonography revealed a 6‐cm pancreatic head mass, prompting referral to our hospital. Contrast‐enhanced CT and magnetic resonance imaging (MRI) identified a 6‐cm mass lesion, with degeneration and calcification extending from the pancreatic head to the tail, along with enlarged periaortic lymph nodes (Figure 1a,b). Given the frequency, solid pseudopapillary neoplasms (SPN) were initially suspected, though pancreatic TB was also considered. Chest CT revealed an old, calcified lesion in the pulmonary hilum. Gastric juice PCR detected *M. tuberculosis*, and interferon‐γ release assay (IGRA) revealed positivity. However, acid‐fast bacilli were absent in smears and cultures of blood, sputum, gastric juice, stool, and cerebrospinal fluid. Fluorodeoxyglucose positron emission tomography displayed faint uptake in the periaortic lymph nodes, suggesting reactive lymphadenopathy without metastases. Cerebral T2‐weighted MRI demonstrated ring‐shaped hyperintensities in the left parietal lobe. Endoscopic ultrasound‐guided fine needle aspiration (EUS‐FNA) of the pancreatic head lesion revealed necrotic tissue with neutrophilic aggregation, indicative of abscess formation (Figure 1c). Ziehl–Neelsen staining, cultures, and PCR were all negative. No caseous necrosis, epithelioid cells, Langhans giant cells, or pseudopapillary structures were identified. Based on the history of latent TB infection, positive IGRA and PCR findings, and absence of malignancy, we provisionally diagnosed pancreatic TB. Cerebral tuberculoma was suspected as the CNS lesion. Given the invasiveness of open biopsy or complete resection, a therapeutic diagnosis was made. Following TB Review Board guidance, treatment with INH, rifampicin (RFP), pyrazinamide (PZA), and ethambutol (EB) was initiated for 2 months, followed by INH, RFP, and EB for 10 months. The pancreatic and CNS lesions resolved, confirming the diagnosis.

**FIGURE 1:**
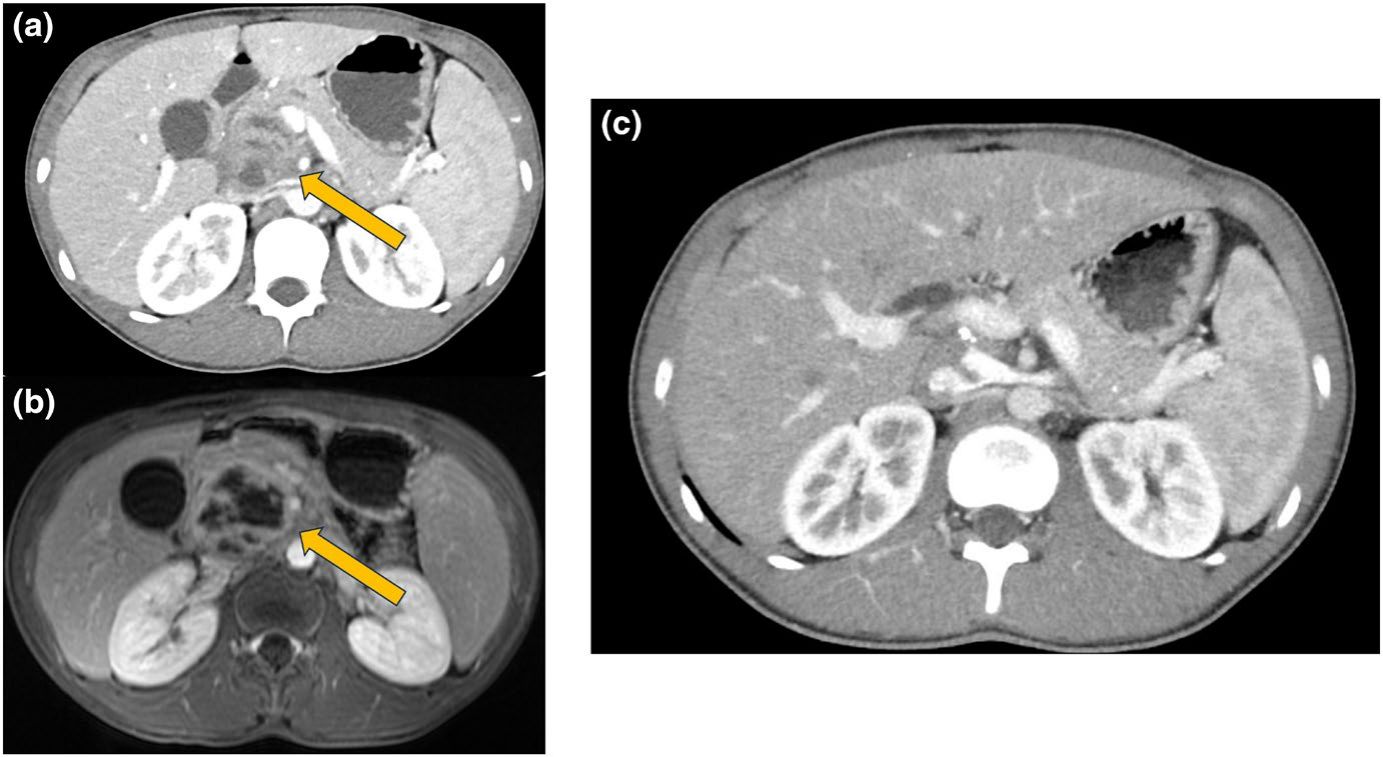
(a) Abdominal contrast‐enhanced CT and (b) T1‐weighted abdominal contrast‐enhanced MRI identified a 6‐cm mass lesion, with degeneration and calcification extending from the pancreatic head to the tail (arrows), along with enlarged periaortic lymph nodes. (c) After treatment, abdominal contrast‐enhanced CT revealed the disappearance of the mass lesion.

Pancreatic TB is generally confirmed postoperatively, with preoperative diagnosis being extremely challenging. In a previous study, 88% (36/41) of cases underwent exploratory laparotomy.^2^ Histology, PCR, smears, and cultures are commonly employed. EUS‐FNA yielded diagnostic findings in 76.2% (16/21) of cases.^3^ PCR has higher sensitivity (64%–75%) and specificity (68%–100%) than cultures or histology, making it particularly useful in abdominal TB.^4^ If EUS‐FNA is inconclusive, surgical intervention may be necessary. In this case, PCR of gastric juice was positive, but culture—generally more sensitive—was negative, possibly due to unneutralized gastric acid after sampling.

Differentiating pancreatic TB from neoplasms is challenging. In young patients, pancreatic neoplasms often include SPN. Radiologically, TB and SPN are indistinguishable.^1^ TB may involve various organs, while SPN metastases are rare and usually confined to the liver.^5^ This case showed paraaortic lymphadenopathy without liver metastases. A favorable therapeutic response confirmed the diagnosis. In patients with a TB history and a pancreatic mass, when imaging and EUS‐FNA are inconclusive, diagnosis may be achieved through response to anti‐TB treatment, avoiding invasive surgery.

## AUTHOR CONTRIBUTIONS

Yuya Nakano, Kenta Yamada, Koji Suzuki, and Motohiro Takeuchi treated the patient and collected data from medical records. Yuya Nakano and Kenta Yamada drafted the manuscript; Motoko Yasutomi and Yusei Ohshima worked on the manuscript. All authors read, critically revised the manuscript, and approved the final manuscript.

## CONFLICT OF INTEREST STATEMENT

The authors declare no conflict of interest.

## ETHICS STATEMENT

Informed consent was obtained from the patient's parents for publication of this case.
